# Intrathoracic gastric volvulus as a severe, delayed surgical complication after left subphrenic peritonectomy and hyperthermic intraperitoneal chemotherapy (HIPEC) for advanced ovarian cancer

**DOI:** 10.1186/1477-7819-11-239

**Published:** 2013-09-23

**Authors:** Roberto Caronna, Paolo Sammartino, Maria Luisa Framarino, Bianca Maria Sollazzo, Roberto Luca Meniconi, Piero Chirletti

**Affiliations:** 1 Department of Surgical Sciences “F. Durante”, Policlinico Umberto I, Viale del Policlinico 155, 00161 Roma, Italy; 2 Department of Surgery “P. Valdoni”, Policlinico Umberto I, Viale del Policlinico 155, 00161 Roma, Italy; 3 Department of Gynecological Obstetrical and Urological Siences, Policlinico Umberto I, Viale del Policlinico 155, 00161 Roma, Italy

**Keywords:** Subphrenic peritonectomy, Diaphragmatic hernia, Cytoreductive surgery, Morbidity, Hyperthermic intraperitoneal chemotherapy (HIPEC)

## Abstract

Patients with extensive peritoneal spread from advanced ovarian cancer often undergo several upper abdominal surgical procedures including subphrenic peritonectomy to obtain optimal cytoreduction. The most common complications are pleural effusions, pancreatic leakage and endoabdominal collections. This case report describes an unusual complication, a diaphragmatic hernia with an intrathoracic gastric volvulus developing four months after the patient underwent left subphrenic peritonectomy during interval debulking surgery and hyperthermic intraperitoneal chemotherapy for advanced ovarian cancer.

## Background

Peritoneal spread is a typical feature in patients with primary advanced or recurrent ovarian cancer. Since the first report by Griffith [1] multiple retrospective series have shown that survival is inversely proportional to residual tumor size [2]. After a meta-analysis showed that maximal cytoreduction improves prognosis [3], the criteria for defining desirable surgical outcome in advanced ovarian cancer (AOC) switched from 'optimal debulking’ with variable residual disease up to 1 to 2 cm in diameter, to microscopic residual disease alone. These findings justified more aggressive surgery including, as in other peritoneal carcinomatoses, hyperthermic intraperitoneal chemotherapy (HIPEC) [4,5]. In patients with extensive peritoneal spread from AOC, bulky upper abdominal disease often precludes optimal cytoreduction thus lowering survival rates [6]. To achieve better cytoreduction rates and improve outcome, centers highly experienced in treating AOC, therefore, now recommend extending standard cytoreduction with extensive upper abdominal surgical procedures, including subphrenic peritonectomy, splenectomy, distal pancreatectomy or tumor stripping from Glisson’s capsule [7-9]. Diaphragmatic surgery includes various procedures, such as subphrenic peritonectomy originally proposed by Sugarbaker (stripping) [10], coagulating minimal lesions less than 5 mm in diameter or, in patients with extensive spread infiltrating the muscle and sometimes the adjacent pleura, full-thickness resection [8]. Diaphragmatic surgery carries its own specific morbidity, mainly including pleural and pulmonary complications (pleural effusions, pneumothorax, pulmonary infections and the need for intrathoracic drainage). Less frequently, it also leads to morbidity related to the other upper abdominal surgical procedures frequently associated with subphrenic peritonectomy, such as splenectomy or distal pancreatectomy leading to pancreatitis, digestive fistulas or abdominal collections [9,11,12]. This case report describing a patient in whom a delayed diaphragmatic hernia manifested after cytoreductive surgery including subphrenic peritonectomy for AOC underlines the need to take this possible late complication related to these multiple upper abdominal surgical procedures into account during postoperative follow-up.

## Case presentation

**Figure 1:**
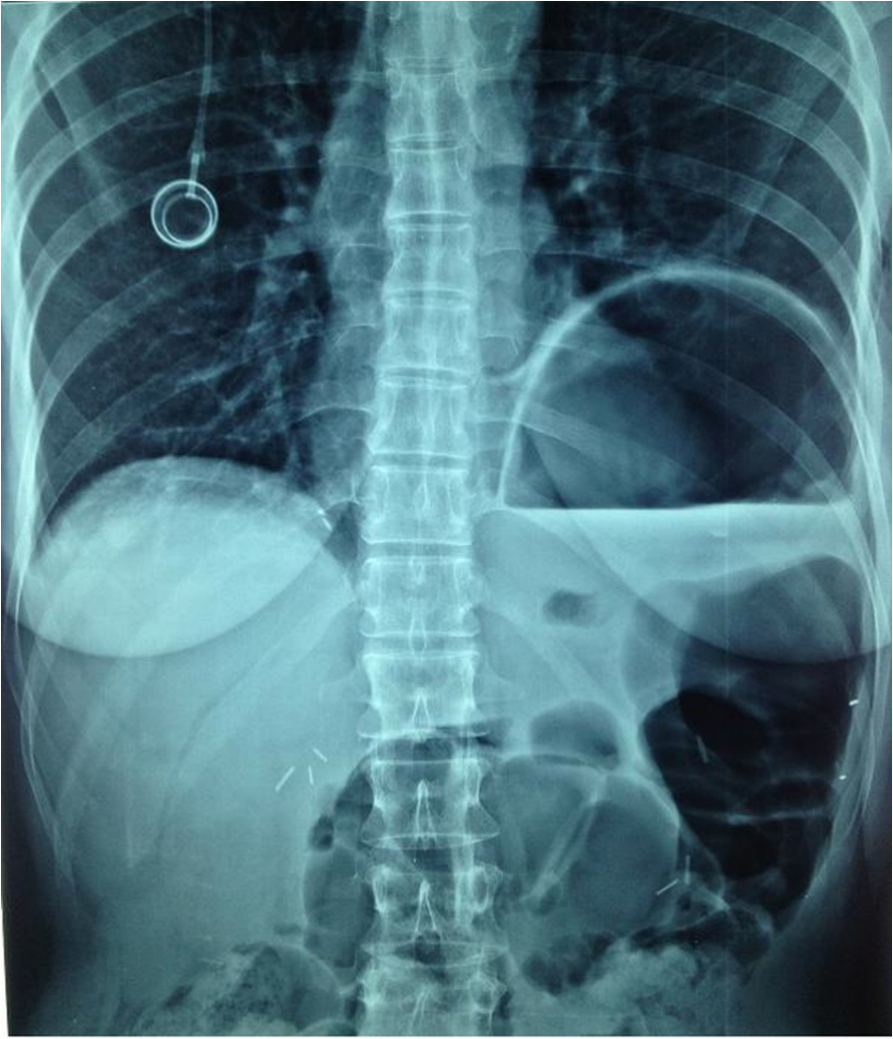
Preoperative plain abdominal X ray showing left hemidiaphragm lifting and gastric overdistension.

**Figure 2:**
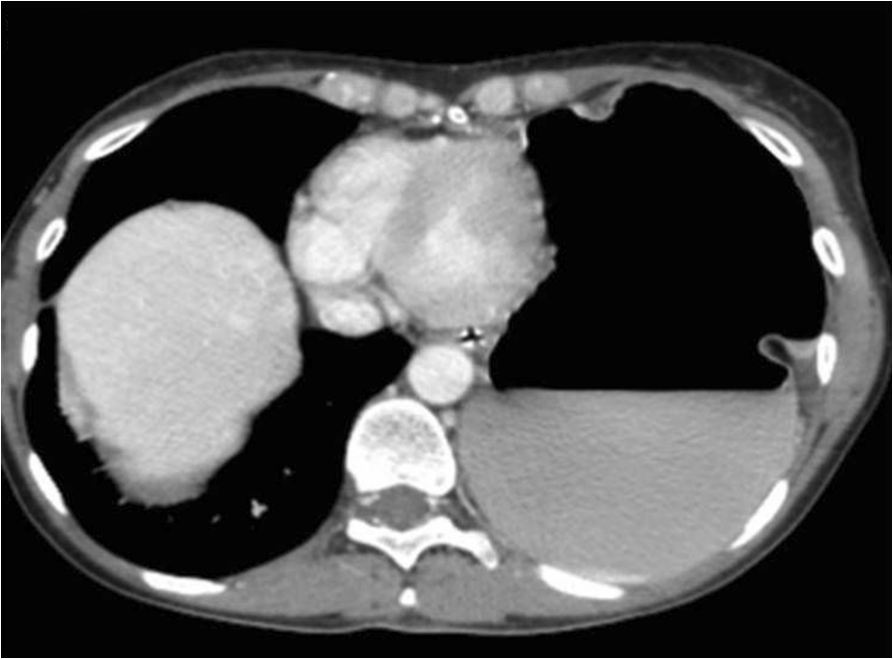
Preoperative plain computed tomographic scan showing left hemidiaphragm lifting and gastric overdistension.

**Figure 3:**
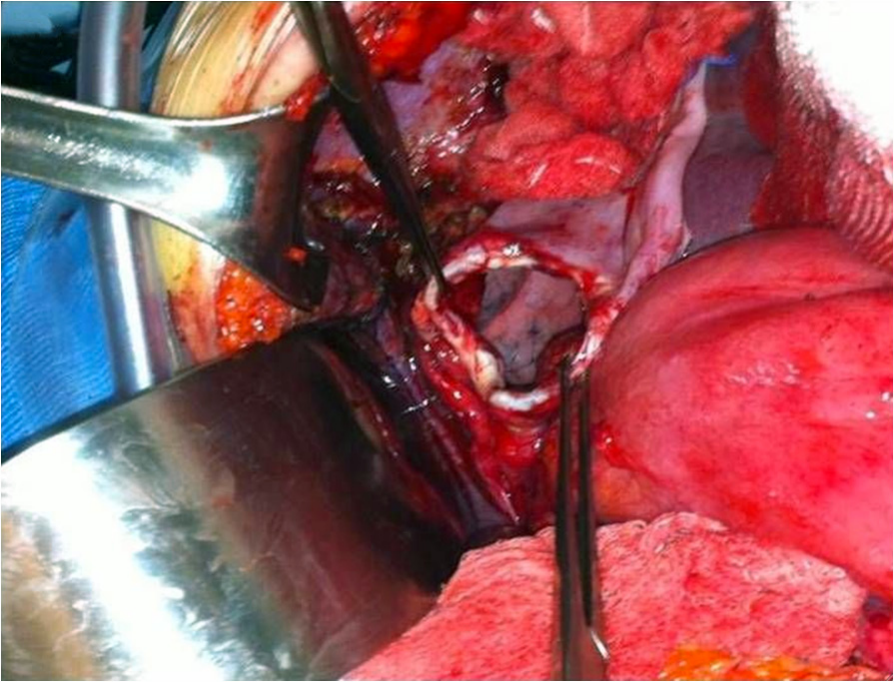
Intraoperative photograph showing the wide breach in the left diaphragm.

A 51-year-old woman was diagnosed with a clinical stage IIIC International Federation of Gynecology and Obstetrics (FIGO) serous ovarian carcinoma in October 2011. Diagnostic laparoscopy showed that debulking surgery as a front-line approach was unlikely to leave no residual tumor so the patient received three courses of paclitaxel and carboplatin neoadjuvant chemotherapy to which she partially responded. When she was hospitalized in January 2012 for interval debulking surgery, intraoperative evaluation showed peritoneal carcinomatosis involving the pelvis, the omentum and the undersurface of both diaphragmatic domes leading to a peritoneal cancer index according to Sugarbaker of 21 [13]. The patient underwent pelvic peritonectomy with bilateral hysteroadnexectomy, appendectomy, cholecystectomy, infragastric omentectomy, bilateral subphrenic peritonectomy and pelvic and lumboaortic lymphadenectomy yielding a completeness of cytoreduction (CC) score of 0 [14]. When the surgical procedure ended, HIPEC was given with a cisplatin solution (75 mg/m^2^) at 43°C for 60 minutes using the closed abdomen technique. Pathological staging classified the tumor as ypT3b pN0 pM0 G3 FIGO stage IIIB. The postoperative course was uneventful and the patient was discharged 11 days after surgery. The patient received consolidation adjuvant chemotherapy with paclitaxel and carboplatin over the next three months and one month later she was readmitted to the hospital with suspected intestinal occlusion. Radiological studies showed left hemidiaphragm lifting and gastric overdistension but no documentable pathological tissue or abdominal fluid collections (Figures 1 and 2). Emergency laparotomy disclosed a wide breach in the left diaphragm with a gastric volvulus herniating into the chest (Figure 3) but no signs indicating recurrent disease. We repositioned the stomach in the abdominal cavity and directly sutured the diaphragmatic breach (no postoperative pleural drainage was necessary). Histological examination of frozen sections from perioperative peritoneal biopsy samples excluded recurrent disease. The postoperative course was uneventful. The patient soon recovered completely and was discharged after a week. She is now under follow-up and clinically disease-free.

## Discussion

A delayed diaphragmatic hernia is an exceptional complication in a patient with AOC who has undergone extensive upper abdominal surgical procedures for maximal cytoreduction. To our knowledge, the literature describes only three similar left-sided cases, given that the liver usually protects the right diaphragm against this event [15,16]. Two of these three cases differ from the case we report here because the clinical features strongly suggest that the diaphragmatic hernia arose for reasons only partially related to diaphragmatic surgery. In the patient described by Laterza *et al*. [15] and in one of the two cases described by Lampl *et al*. [16], the colonic fistula that developed in the splenic flexure after cytoreductive surgery caused an abdominal abscess that invaded through the diaphragm into the chest. Equally important, cytoreduction included a left subphrenic peritonectomy only in the patient described by Laterza *et al*. [15]. In the patient we describe here, as in the second patient reported by Lampl *et al*. [16], the diaphragmatic hernias both had a delayed onset after cytoreductive surgery including subphrenic peritonectomy and were both unrelated to other surgical complications. The only difference concerned the clinical onset because in our patient the ruptured diaphragm caused an intrathoracic gastric volvulus necessitating emergency surgery whereas in the case reported by Lampl *et al*. [16], although computed tomographic (CT) scans obtained during follow-up showed the diaphragmatic hernia progressing over time; only when the symptoms and dyspepsia increased did the patient undergo surgery. What events cause a diaphragmatic hernia to complicate cytoreductive surgery including subphrenic peritonectomy and HIPEC remains conjectural. The clinical history suggests that in the case we report several causes probably acted in concert. First, peritoneal malignant implants involving the diaphragm presumably injured the muscle and the resulting trauma then continued when the patient underwent neoadjuvant chemotherapy followed by surgery. Necrosis in response to neoadjuvant chemotherapy, as others have reported after diaphragmatic surgical procedures done for interval debulking surgery [12], might play a role in increasing the complication rate. In our patient, the subphrenic peritonectomy done during debulking could then have aggravated the previous diaphragmatic trauma when the high-voltage stripping knife induced a thermal shock. Last, HIPEC done even for a short time with the closed abdomen technique caused endoabdominal pressure to increase, thus further traumatizing the diaphragmatic muscle. In our opinion, no specific measures can prevent this infrequent complication after cytoreductive surgery including subphrenic peritonectomy except, possibly, carefully checking intraoperatively that surgery leaves the diaphragm intact. As a pathogenetic explanation, we suggest that our patient’s delayed diaphragmatic hernia, like those sometimes developing after blunt trauma [17], became clinically manifest only when the diaphragmatic trauma devitalized the muscle and caused peripheral denervation, thus progressively thinning the muscle wall and inducing fibrosis until the diaphragm ruptured and the complication turned into a clinical urgency.

## Conclusions

Our case report serves as a useful reminder that physicians following patients with AOC who undergo a subphrenic peritonectomy, especially patients who have received neoadjuvant chemotherapy, should keep this possible complication in mind. The only possible way to prevent this almost unpredictable event is to check that small accidental diaphragmatic perforations left after peritoneal stripping are not overlooked and remain unsutured.

## Consent

“Written informed consent was obtained from the patient for the publication of this report and any accompanying images”.

## Abbreviations

AOC: Advanced ovarian cancer; HIPEC: Hyperthermic intraperitoneal chemotherapy.

## Competing interests

The authors declare they have no competing interests.

## Authors’ contributions

All authors contributed equally to conception and design of the study, analyzing and interpreting data, drafting and revising the manuscript. All authors read and approved the final manuscript.

## References

[B1] Griffith CT Surgical resections of tumour bulk in the primary treatment of ovarian carcinoma NCI Monogr 1975 42 101 104 1234624

[B2] Elattar A Bryant A Winter-Roach BA Hatem M Naik R Optimal primary surgical treatment for advanced epithelial ovarian cancer Cochrane Database Syst Rev 2011 8 CD007565 21833960 10.1002/14651858.CD007565.pub2 PMC6457688

[B3] Bristow RE Tomacruz RS Armstrong DK Trimble EL Montz FJ Survival effect of maximal cytoreductive surgery for advanced ovarian carcinoma during the platinum era: a meta-analysis J Clin Oncol 2002 20 1248 1259 10.1200/JCO.20.5.1248 11870167

[B4] du Bois A Reuss A Pujade-Luraine E Harter P Ray-Coquard I Pfisterer J Role of surgical outcome as prognostic factor in advanced epithelial ovarian cancer: a combined exploratory analysis of 3 prospectively randomized phase 3 multicentric trials by the Arbeitsgemeinschaft Gynaekologische Onkologie Studiengruppe Ovarialkarzinom (AGO-OVAR) and the Groupe d’Investigateurs Nationaux pour les Etudes des Cancers de l’ovaire (GINECO) Cancer 2009 115 1234 1244 10.1002/cncr.24149 19189349

[B5] de Bree E Helm CW Hyperthermic intraperitoneal chemotherapy in ovarian cancer: rationale and clinical data Expert Rev Anticancer Ther 2012 12 895 911 10.1586/era.12.72 22845405

[B6] Chi DS Franklin CC Levine DA Akselrod F Sabbatini P Jarnagin WR DeMatteo R Poynor EA Abu-Rustum NR Barakat RR Improved optimal cytoreduction rates for stages IIIC and IV epithelial ovarian, fallopian tube, and primary peritoneal cancer: a change in surgical approach Gynecol Oncol 2004 94 650 654 10.1016/j.ygyno.2004.01.029 15350354

[B7] Eisenhauer EL Abu-Rustum NR Sonoda Y Levine DA Poynor EA Aghajanian C Jarnagin WR DeMatteo RP D'Angelica MI Barakat RR Chi DS The addition of extensive upper abdominal surgery to achieve optimal cytoreduction improves survival in patients with stages IIIC-IV epithelial ovarian cancer Gynecol Oncol 2006 103 1083 1090 10.1016/j.ygyno.2006.06.028 16890277

[B8] Tsolakidis D Amant F Van Gorp T Leunen K Neven P Vergote I Diaphragmatic surgery during primary debulking in 89 patients with stage IIIB-IV epithelial ovarian cancer Gynecol Oncol 2010 116 480 496 10.1016/j.ygyno.2009.07.014 19954825

[B9] Chéreau E Rouzier R Gouy S Ferron G Narducci F Bergzoll C Huchon C Lécuru F Pomel C Daraï E Leblanc E Querleu D Morice P Morbidity of diaphragmatic surgery for advanced ovarian cancer: retrospective study of 148 cases Eur J Surg Oncol 2011 37 175 180 10.1016/j.ejso.2010.10.004 21093204

[B10] Sugarbaker PH Peritonectomy procedures Ann Surg 1995 221 29 42 10.1097/00000658-199501000-00004 7826158 PMC1234492

[B11] Chi DS Zivanovic O Levinson KL Kolev V Huh J Dottino J Gardner GJ Leitao MM Jr Levine DA Sonoda Y Abu-Rustum NR Brown CL Barakat RR The incidence of major complications after the performance of extensive upper abdominal surgical procedures during primary cytoreduction of advanced ovarian, tubal and peritoneal carcinomas Gynecol Oncol 2010 119 38 42 10.1016/j.ygyno.2010.05.031 20609464

[B12] Terauchi F Okamoto A Wada Y Hasegawa E Sasaki T Akutagawa O Sagawa Y Nishi H Isaka K Incidental events of diaphragmatic surgery in 82 patients with advanced ovarian, primary peritoneal and fallopian tubal cancer Oncol Letters 2010 1 861 864 10.3892/ol_00000152 PMC3436412 22966395

[B13] Jacquet P Sugarbaker PH Sugarbaker PH Clinical research methodologies in diagnosis and staging of patients with peritoneal carcinomatosis Peritoneal Carcinomatosis: Principles of Management 1996 Boston: Kluwer 359 374 10.1007/978-1-4613-1247-5_23 8849962

[B14] Sugarbaker PH Peritoneal Carcinomatosis: Principles of Management 1996 Boston: Kluwer

[B15] Laterza B Baratti D Cozzi G Kusamura S Oliva GD Gavazzi C Fumagalli L Sironi A Sabia D Deraco M Colobronchial fistula: an unusual complication after peritonectomy and hyperthermic intraperitoneal chemotherapy (HIPEC) In vivo 2009 23 151 154 19368141

[B16] Lampl B Leebmann H Mayr M Piso P Rare diaphragmatic complications following cytoreductive surgery and HIPEC: report of two cases Surg Todayin press 10.1007/s00595-012-0445-9 23224234

[B17] Rashid F Chakrabarty MM Singh R Iftikhar SY A review on delayed presentation of diaphragmatic rupture World J Emerg Surg 2009 4 32 10.1186/1749-7922-4-32 19698091 PMC2739847

